# Correction to: Factors Affecting Home Injuries in Older Adults: An Analysis Using Binary Logistic Regression

**DOI:** 10.1002/hsr2.71681

**Published:** 2025-12-21

1

Request for a **Corrigendum** in the Published Article

Dear Editor‐in‐Chief, Dr Qian Chen

In the following published paper:

**Title: Factors Affecting Home Injuries in Older Adults: An Analysis Using Binary Logistic Regression.** Health Science Reports. 2025 Jul 15; 8(7):e71055. doi: 10.1002/hsr2.71055

In the **Methods, Measurement Tools section (page 3 of 12)**, the instrument used to assess Activities of Daily Living should be replaced as follow:

**“**2.4.3 | **Activities of Daily Living (ADL)**

Independence in daily activities was measured using a modified Activities of Daily Living (ADL) scale, originally developed by Taheri et al. [40]. The modified instrument assessed bowel and bladder incontinence as distinct items and utilized the transferring item to measure stepping. Item‐level responses were coded as 0 (dependent), 1 (needs help), or 2 (independent). Summed scores (range: 0–16) were used to classify participants into one of three functional categories: dependent (0–7), requires assistance (8–11), or independent (12–16). The scale's validity for the Iranian older adult population has been established, and its reliability in the present study was confirmed with a Cronbach's α of 0.80.**”**

**Instead of the current explanation:**

“2.4.3 | ADL (Activities of Daily Living), KATZ

The ADL index assesses individuals based on their level of independence in performing daily activities. Each item is scored as dependent (0 points), needs help (1 point), or independent (2 points). The overall score ranges from 0 to 16, and individuals are categorized as dependent (0– 7 points), needing help (8–11points), or independent (12–16 points). This scale has been validated for use among older individuals in Iran. Total Cronbach's α coefficient was 0.80 [40].”

This change is strictly terminological in nature and has no bearing on the study's data, scoring, analytical methods, results, or conclusions.

We kindly request the publication of a Corrigendum to reflect this correction and ensure the accuracy of the scientific record.

Kind regards,

***Prof. Hamid Allahverdipour (Corresponding Author)***

*Head of Iranian Health Education and Promotion Association*

*Secretary of the Board of Health Education and Promotion, Ministry of Health, Iran*

*Editor‐in‐Chief of Health Promotion Perspectives*

*Tabriz University of Medical Sciences*

*Tabriz, 14711‐IRAN*

*email 1: allahverdipourh@tbzmed.ac.ir*

*email 2: allahverdipour@gmail.com*

*Tel & Fax: +98‐41‐333444731*

